# Gender Differences in Demographic and Pharmacological Factors in Patients Diagnosed with Late-Onset of Alzheimer’s Disease

**DOI:** 10.3390/brainsci12020160

**Published:** 2022-01-26

**Authors:** Melissa J. Bailey-Taylor, Nicolas Poupore, Laurie Theriot Roley, Richard L. Goodwin, Brooks Mcphail, Thomas I. Nathaniel

**Affiliations:** 1 PRISMA Health Upstate, Greenville, SC 29605, USA; melissa.bailey4@prismahealth.org (M.J.B.-T.); Laurie.Theriot@prismahealth.org (L.T.R.); 2 University of South Carolina School of Medicine Greenville, 607 Grove Rd, Greenville, SC 29605, USA; npoupore@email.sc.edu (N.P.); GOODWIRL@greenvillemed.sc.edu (R.L.G.); bmcphail@greenvillemed.sc.edu (B.M.)

**Keywords:** cholinesterase inhibitors, early-onset Alzheimer’s disease, late-onset Alzheimer’s disease

## Abstract

Background: Whether gender differences exist in late-onset of Alzheimer’s disease (LOAD) treated with cholinesterase inhibitors (ChEIs) is not fully understood. This study investigated demographic and pharmacological characteristics in LOAD patients to determine gender differences in LOAD patients treated with ChEIs alone and ChEIs with other medications. Methods: This 5-year retrospective data analysis included 9290 LOAD AD patients with 2949 men patients and 6341 women. Potential predictors of demographic and pharmacological characteristics associated gender differences in patients treated with and without ChEIs therapy were determined using univariate analysis, while multivariable models adjusted for demographic and pharmacological variables. Results: In the adjusted analysis, men patients with LOAD that presented with a history of alcohol use (ETOH) (OR = 1.339, 95% CI, 1.072–1.672, *p* = 0.010), treated with second generation antipsychotics (SGAs) (OR = 1.271, 95% CI, 1.003–1.610, *p* = 0.047), citalopram (OR = 5.103, 95% CI, 3.423–7.607, *p* < 0.001), memantine (OR = 4.409, 95% CI, 3.704–5.249, *p* < 0.001), and buspirone (OR = 2.166, 95% CI, 1.437–3.264, *p* < 0.001) were more likely to receive ChEIs therapy, whereas older men were less likely to be treated with ChEIs therapy. Women who were African Americans (OR = 1.387, 95% CI, 1.168–1.647, *p* < 0.001), that received memantine (OR = 3.412, 95% CI, 3.034–3.837, *p* < 0.001), selective serotonin reuptake inhibitor (SSRIs) (OR = 1.143, 95% CI, 1.016–1.287, *p* = 0.026), and a history of ETOH (OR = 2.109, 95% CI, 1.724–2.580, *p* < 0.001) were more likely to receive ChEIs therapy, whereas older women were less likely to receive ChEIs therapy. Conclusion: In both men and women patients, those with increasing age were less likely to be treated with ChEI therapy, while patients treated with memantine were also likely to receive ChEI therapy. Our findings highlight the importance for clinicians to optimize ChEI in LOAD to improve treatment effectiveness and eliminate gender differences in ChEI therapy.

## 1. Introduction

Several studies [1,2,3,4] indicate that more than two-thirds of clinically diagnosed cases of dementia and Alzheimer’s disease (AD) are women. The observed gender difference is linked to women’s greater longevity, especially since the risk of developing dementia increases with age [5]. The extent to which gender differences are associated with women relative to men at older ages or also of women’s having a greater risk than men at the same age has been investigated in different studies [5,6,7,8,9,10]. The higher number of AD cases reported among women tend to indicate that women present with a higher incidence of AD when compared with men [5,6,7]. However, a deeper review of the existing literature indicates that the observed gender differences in AD are more complex than currently being reported. Several studies that found that gender differences [8,9,10] tend to show later in life, and the variation point at which incidence rates begin to differ between men and women also vary in different studies.

Psychiatric and behavioral symptoms are common in patients with AD [11]. The effect of second-generation antipsychotics (SGAs) is known to be associated with improvement in psychiatric symptoms, more specifically agitation, hostile suspiciousness, and psychotic distortion [12]. While several studies found that clinical symptoms such as anger, aggression, and paranoid ideas improved with SGA treatment [13,14], whether there is evidence of gender difference in the treatment with SGAs is not fully understood. While cholinergic dysfunction was long thought to be the only contributor to AD symptomatology [15], growing evidence supports a critical role for the serotonergic system in memory retention and learning by interacting with the cholinergic dopaminergic, γ-aminobutyric acid (GABA)ergic, and glutaminergic systems [16]. Selective serotonin reuptake inhibitors (SSRIs) are approved as first-line treatment of depressive disorders and with less severe effect profiles when compared to older antidepressants such as the tricyclics [17]. SSRIs function by selectively targeting the solute carrier family 6 member 4 responsible for terminating the action of serotonin in the synaptic cleft, consequently increasing this neurotransmitter availability at the synapse [18]. Due to women’s longer life expectancy or sociocultural detection bias [5]; the progression of the diseases is reported to be more rapid among elderly women [19]. While SSRIs have emerged as promising strategies to delay the onset of AD in both men and women, it is not clear whether there is evidence of gender difference in treatment with SSRIs among LOAD patients.

While most studies suggest that approximately 80 years old is the age in which incidence rates in women either shift position with or increase above men’s rates [9,20,21,22], there are some notable exceptions. For example, studies have reported incidence rates that begin to deviate as early as 75–79 years [23] and as late as 90 in patients who present with LOAD [24]. Diagnosis of AD before the age of 65 years are described as presenting with early-onset Alzheimer’s disease (EOAD) [25], while those with an onset of AD after 65 years of age are described as having LOAD [26]. The prevalence of patients with EOAD is reported to be low but varies from 6.0% to 16.5% [27,28]. In terms of cognition, patients with EOAD demonstrated more impairment in language and concentration, whereas LOAD patients presented difficulties in memory and orientation [29].

Currently, the main therapy for AD ranging from mild to severe AD are the cholinesterase inhibitors (ChEIs) [30,31]. Positive cognitive and global symptomatic effects of ChEI therapy have been reported in a randomized clinical trial [32] and observational studies [33,34,35] for both EOAD and LOAD [5]. While LOAD has been reported to present more functional deficits and severities than the EOAD [36], possible differences in demographic and other pharmacological factors in patients treated with and without ChEIs, and how this might contribute to a gender difference in a cohort with exclusively LOAD is not fully understood. This issue was investigated in the current study.

A short-term positive cognitive response to ChEIs in men when compared with women was reported for tacrine and galantamine [37,38]. The observed difference was attributed to the role of sex hormones in AD [39], and larger cerebral hemispheres observed in men even after adjusting for the effect of body size [40]. In a similar study, the relationship between AD pathology and dementia was higher in women than in men [5], and men were reported to present with less functional deficits when compared with women [6]. This explanation was provided to explain the more favorable cognitive outcome and the better response for men following treatment with ChEIs and other medications including SSRIs and SGAs. Whether such differences can be associated with the demographic and pharmacological characteristics of LOAD treated with ChEIs alone and ChEIs with other pharmacological medications such as SSRIs and SGAs is yet to be investigated. Therefore, in this study, we sought to explore gender differences in LOAD that received and did not receive ChEIs. We compared men and women by looking at demographic and pharmacological characteristics to determine whether men and women patients with LOAD were treated with ChEIs alone or ChEIs along with other medications. Because our sample was restricted to LOAD with dementia patients that present moderate to severe symptoms, we assumed that more women than men may be affected, which is typical for the AD population. Therefore, we hypothesized that men and women LOAD patients would differ in regard to treatment with ChEIs. Therefore, our first objective is to determine whether there are differences in men and women LOAD patients treated with ChEI therapy. Moreover, since men and women present with differences in cognitive and functional progression with women declining at much higher rates than men [8,10], our second objective is to determine specific demographic and pharmacological factors that contribute to gender differences in patients that received ChEIs. The aim of the current study is to contribute to the existing literature of LOAD with ChEIs therapy to evaluate whether ChEI therapy and ChEI combined with other therapies are higher in women than men. Using a retrospective registry offers several benefits, including a large sample size from a single data base; the ability to link to administrative records; and the application of uniform methodology to identify ChEI therapy with other pharmacological characteristics for men and women across LOAD patients.

## 2. Methods

### 2.1. Study Population

This study was approved by the Institutional Review Board (IRB) of PRISMA Health. We retrospectively analyzed data for patients who presented to Prisma Health-Upstate (formerly known as Greenville Health System) with the diagnosis of Alzheimer’s Disease from February 2016 to July 2020. Patients may have presented due a complaint stemming from their AD or for an unrelated problem. Most patients presented to a Prisma Health-Upstate Emergency Department, but patients also were recorded who presented to other locations, like an operating room, or gastrointestinal laboratory. Patients with AD with behavioral disturbances (e.g., anxiety, irritation, mood changes, verbal and physical aggression, wandering, or agitation), without behavioral disturbances, and unknown status of behavioral disturbances were all included in this study. Patients were excluded if they were diagnosed with EOAD or presented with AD under the age of 65.

Demographics, medication history, and social risk factors were all were collected from a comprehensive database. The demographic variables included age, race/ethnicity, gender, and ethnicity. Social risk factors included alcohol use and tobacco use history. Alcohol use was based on if a patient reported that they ever consumed alcohol, regardless of the amount of time that had passed, and tobacco use was categorized in a similar fashion. Medication history was recorded for mainly central nervous system agents. These ChEIs, specifically donepezil, galantamine, and rivastigmine, second generation antipsychotics (SGA), specifically aripiprazole, olanzapine, and risperidone, and selective serotonin receptor inhibitors (SSRI), specifically citalopram, escitalopram, and paroxetine. Memantine, trazadone, buspirone, and valproate usage were also included in our analysis. Dosages of medications were not recorded in the data base. 

For the diagnosis, following clinical diagnosis of memory loss following a brief initial screening test using Item Informant Questionnaire was provided [41]. This allows the general assessment of cognition, and the ascertainment of dementia using the Diagnostic and Statistical Manual of Mental Disorders, 5th ed. with updated diagnostic criteria [42]. The screening tests allowed for a quick assessment and the need for further evaluation [37]. Following a positive assessment, a subsequent cognitive evaluation using the Mini-Mental State Examination, Montreal Cognitive Assessment, Saint Louis University Mental Status Examination [43] as the screening tool was performed. Other follow-up evaluation includes screening for depression, performance of laboratory testing and ordering structural neuroimaging [44].

### 2.2. Statistical Analysis

The initial analysis focused on performing univariate statistical analysis to determine demographic and pharmacological characteristics of patients with LOAD by gender. The normal distribution of our data was tested using a Kolmogorov–Smirnov test following the determination of mean and standard deviation. In addition, our data was also validated for normal distribution using Lilliefors test that gives more accurate results. Descriptive statistics was used, and a Student’s *t*-test was considered for continuous variables while a Man–Whitney-U or Pearson chi-square test as appropriate was used to analyze discrete variables to determine comparisons between the ChEI and non ChEI group. Similar analysis was performed to determine different demographic and pharmacological risk factors in patients with late LOAD who were or were not taking a ChEI, stratified by gender. For all continuous variables, the mean, standard deviation, and range were calculated and for all discrete variables, the number of patients and percentage of patients in that category were determined. Then, the logistic models were built using the established predictors from the second univariate analysis. This post hoc adjusted analysis was executed using the likelihood ratio backward selection method. This method was chosen because all the initially selected demographic, social, and pharmacological risk factors were included in the model and then systematically removed if they did not contribute to the overall significance of the model.

For the multivariate analysis, multicollinearity was determined for the interactive effects of variables using variance inflation factors (VIFs). In addition, we tested the validity of our model using a Hosmer–Lemeshow test, while the overall correct classification percentage and the area under the receiver operating curve (AUROC) for score prediction was determined to test the sensitivity, specificity, and accuracy of the model. For each of the regression models, the dependent variable was the presence of ChEI therapy. The primary independent variables were the demographic, social, and pharmacologic factors stratified by gender in patients diagnosed with LOAD. Odds ratios and 95% confidence intervals (95% CIs) of outcome measures were obtained from this model with the significance of 0.05. The odds of receiving ChEI therapy for LOAD were determined separately for men, women, and the entire sample independent of gender. The odds ratios values were used to determine independent variables that predicted LOAD patients by gender that received ChEI therapy. The overall correct classification percentage and area under the Receiver Operating Curve (ROC) were used to determine the sensitivity, specificity, and accuracy of the logistic regression model for all three groups (whole LOAD population, men and women LOAD that had received or had not received ChEI therapy alone and/or combined with other medications). All statistical analyses were done using the Statistical Package for Social Sciences v 26.0 for Windows (SPSS, Chicago, IL, USA).

## 3. Results

A total of 9290 of LOAD patients were identified in this study. Of this population, 2949 patients were men and 6341 were women (Table 1). As shown in Table 1, women were likely to be older (86.60 ± 7.51 vs. 85.59 ± 7.15, *p* < 0.001), from Hispanic ethnic group (2.3% vs. 1.1%, *p* < 0.001), but less likely to be white or Caucasians (87.8% vs. 82.3%, *p* < 0.001). Fewer women presented with a history of ETOH use (10.9% vs. 18.2%), and tobacco use (32.5% vs. 65.5%, *p* < 0.001), and tobacco use (32.5% vs. 65.5%, *p* < 0.001). Women were less likely to be taking galantamine (1.2% vs. 2.1%, *p* < 0.001), and memantine (43.9% vs. 47.0%, *p* < 0.001), but more likely to be on an SSRI (35.6% vs. 27.3%, *p* < 0.001), specifically citalopram (12.9% vs. 9.7%, *p* < 0.001), escitalopram (23.5% vs. 17.9%, *p* < 0.001), aripiprazole (2.8% vs. 1.2%, *p* < 0.001), and buspirone (7.9% vs. 5.8%).

The demographic and pharmacological characteristics associated with ChEI therapy in LOAD patients stratified by gender is presented in Table 2. In the men group, 1047 did not receive ChEI therapy while 1902 received ChEI therapy. Men that received ChEI were younger (85.26 ± 7.07 vs. 86.21 ± 7.24, *p* < 0.001), and presented with a history of ETOH use (19.9% vs. 15.1%, *p* < 0.002). This group also presented with a higher usage of SGA (17.4% vs. 13.4%, *p* < 0.005), SSRIs (32.3% vs. 18.4%, *p* < 0.001), specifically citalopram (13.4% vs. 3.1%, *p* < 0.001), escitalopram (19.3% vs. 15.4%, *p* < 0.001), memantine (59.5% vs. 24.3%, *p* < 0.001), and buspirone (7.2% vs. 3.2%, *p* < 0.001). Women who received ChEIs were more likely to be younger (85.86 ± 7.18 vs. 87.85 ± 7.88, *p* < 0.001), less likely to be Caucasians (81.3% vs. 83.9%, *p* < 0.001), and presented with higher rates of ETOH use (13.3% vs. 6.7%, *p* < 0.001). This group was more likely to be taking a SSRI (37.8% vs. 31.9%, *p* < 0.001), specifically escitalopram (25.2% vs. 20.7%, *p* < 0.001), and memantine (55.1% vs. 25.0%, *p* < 0.001).

**Figure 1:**
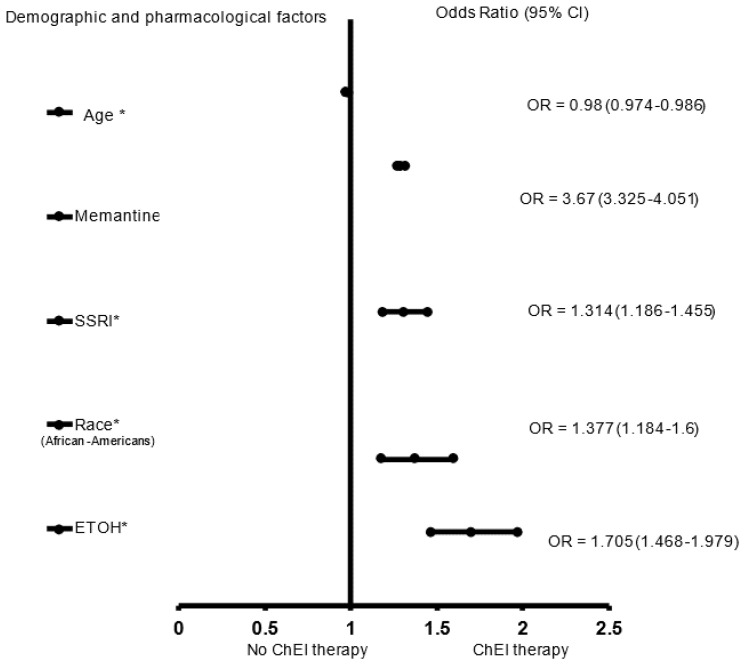
The forest plot shows the demographics and pharmacological characteristics associated with cholinesterase inhibitor (ChEI) for the whole late onset Alzheimer’s disease patient population. Adjusted OR < 1 denote factors that are associated with not receiving ChEI while OR > 1 denote factors that are associated with receiving ChEI. Hosmer_Lemeshow test (*p* < 0.001 *), Cox and Snell (*R*^2^ = 0.107). The overall percentage of 67.2% was applied to check for fitness of the logistic regression model. * Indicates statistical significance (*p* < 0.05) with a 95% confidence interval.

**Figure 2:**
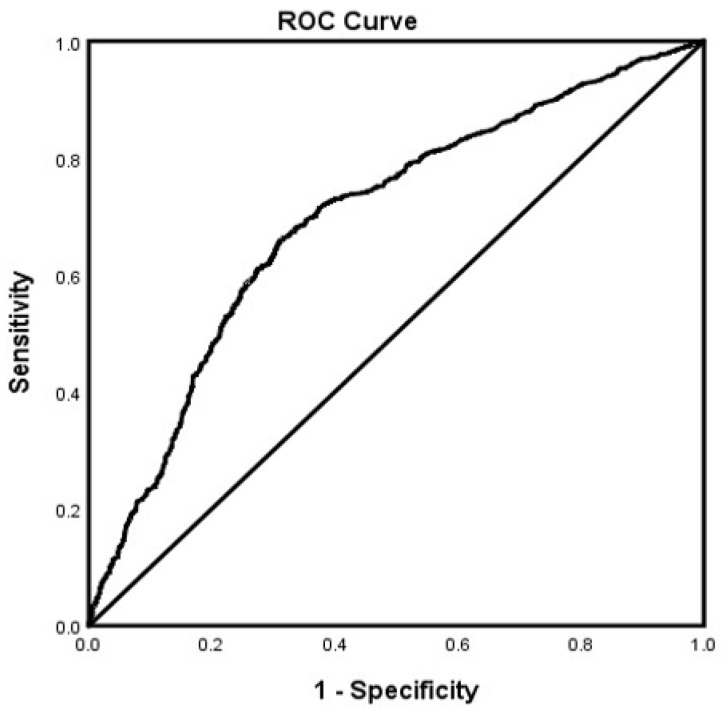
ROC curve associated with prediction of receiving cholinesterase inhibitor (ChEI) for late onset Alzheimer’s patients independent of gender. Higher area under the curve (AUC) values in ROC analysis indicate better discrimination of the score for the measured outcome. Classification table (overall correctly classified percentage = 67.2%) and area under the ROC curve (AUC = 0.697, 0.685–0.708) were applied to check model fitness.

**Figure 3:**
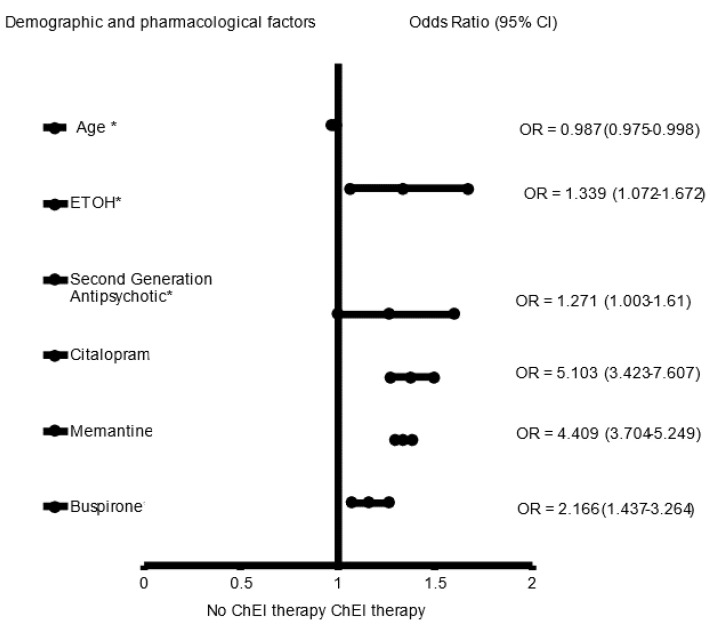
The forest plot for the demographics and pharmacological characteristics associated with cholinesterase inhibitor (ChEI) for the men late onset Alzheimer’s disease patient population. Adjusted OR < 1 denote factors that are associated with not receiving ChEI while OR > 1 denote factors that are associated with receiving ChEI. Hosmer_Lemeshow test (*p* < 0.001 *), Cox and Snell (*R*^2^ = 0.150). The overall classified percentage of 70.1% was applied to check for fitness of the logistic regression model. * Indicates statistical significance (*p* < 0.05) with a 95% confidence interval.

**Figure 4:**
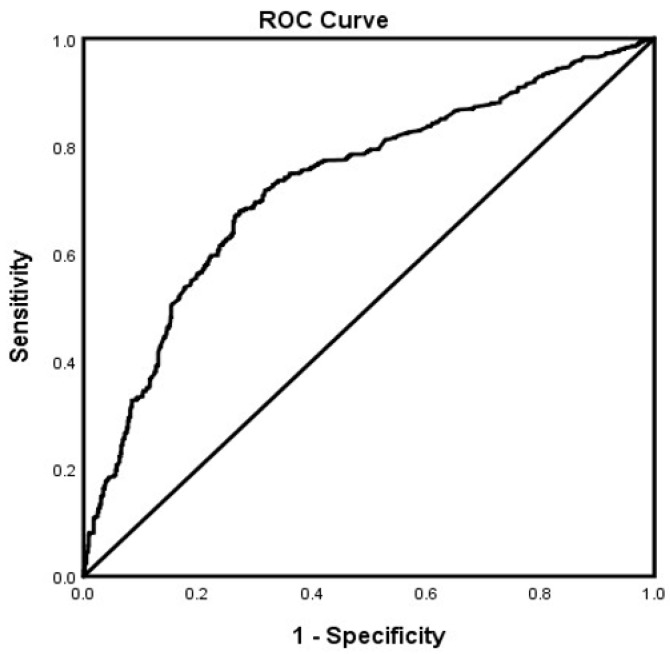
ROC curve associated with prediction of receiving cholinesterase inhibitor (ChEI) for men late onset Alzheimer’s patients. Higher area under the curve (AUC) values in ROC analysis indicate better discrimination of the score for the measured outcome. Classification table (overall correctly classified percentage = 70.1%) and area under the ROC curve (AUC = 0.730, 0.711–0.749) were applied to check model fitness.

**Figure 5:**
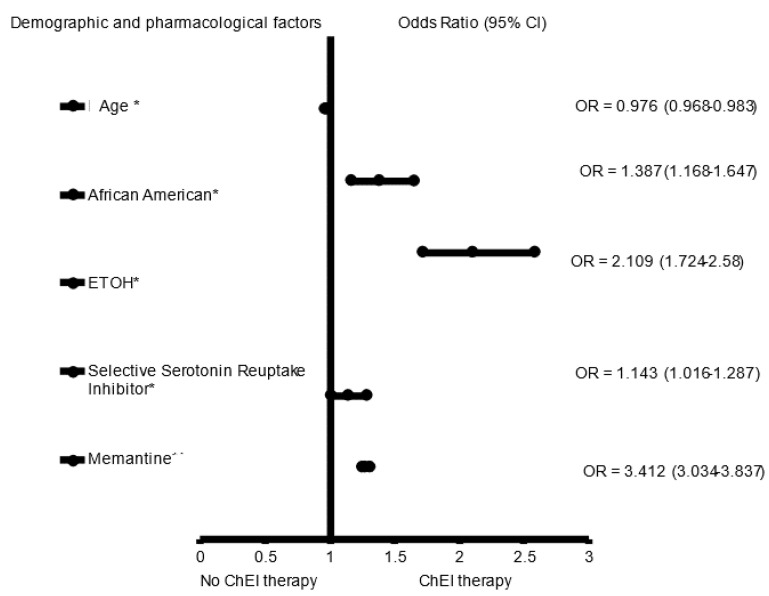
The forest plot for the demographics and pharmacological characteristics associated with cholinesterase inhibitor (ChEI) for the women late onset Alzheimer’s disease patient population. Adjusted OR < 1 denote factors that are associated with not receiving ChEI while OR > 1 denote factors that are associated with receiving ChEI. Hosmer_Lemeshow test (*p* < 0.001 *), Cox and Snell (*R*^2^ = 0.103). The overall classified percentage of 66.6% was applied to check for fitness of the logistic regression model. * Indicates statistical significance (*p* < 0.05) with a 95% confidence interval.

**Figure 6:**
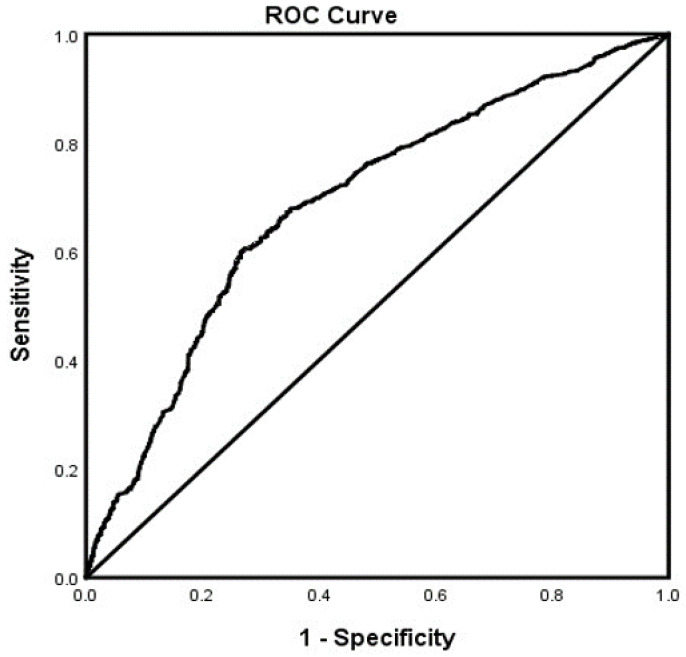
ROC curve associated with prediction of receiving cholinesterase inhibitor (ChEI) for women late onset Alzheimer’s disease patients. Higher area under the curve (AUC) values in ROC analysis indicate better discrimination of the score for the measured outcome. Classification table (overall correctly classified percentage = 66.6%) and area under the ROC curve (AUC = 0.688, 0.674–0.702) were applied to check model fitness.

In the adjusted analysis, memantine (OR = 3.670, 95% CI, 3.325–4.051, *p* < 0.001), SSRIs (OR = 1.314, 95% CI, 1.025.–1.119, *p* = 0.033), African Americans (OR = 1.377, 95% CI, 1.184–1.600, *p* < 0.001), and a history of ETOH (OR = 1.705, 95% CI, 1.468–1.979, *p* < 0.001) were associated with receiving ChEI in all patients with LOAD independent of gender, while older people (OR = 0.980, 95% CI, 0.974–0.986, *p* < 0.001) were less likely to receive ChEI therapy (Figure 1). The ROC curve for the predictive power of the regression model is presented in Figure 2. The discriminating capability of the model was moderately strong as shown by the ROC curve, with area under the curve (AUROC) = 0.697 (95% CI, 0.685–0.708, *p* < 0.001). The forest plot for the logistic regression model for the men with LOAD is presented in Figure 3. As shown in the figure, history of ETOH (OR = 1.339, 95% CI, 1.072–1.672, *p* = 0.010), SGAs (OR = 1.271, 95% CI, 1.003–1.610, *p* = 0.047), citalopram (OR = 5.103, 95% CI, 3.423–7.607, *p* < 0.001), memantine (OR = 4.409, 95% CI, 3.704–5.249, *p* < 0.001), and buspirone (OR = 2.166, 95% CI, 1.437–3.264, *p* < 0.001) were more likely to be associated with ChEIs therapy, whereas older people (OR = 0.987, 95% CI, 0.975–0.998, *p* = 0.027) were less likely to receive ChEI therapy. As presented in Figure 4, the predictive power of the logistic regression was moderately strong. The area under the curve (AUROC) is 0.730 (95% CI, 0.711–0.749, *p* < 0.001). The forest plot representation of the logistic regression model for women patients with LOAD with or without ChEI therapy is presented in Figure 5. As shown in the figure, women African American patients (OR = 1.387, 95% CI, 1.168–1.647, *p* < 0.001), treated with memantine (OR = 3.412, 95% CI, 3.034–3.837, *p* < 0.001), SSRIs (OR = 1.143, 95% CI, 1.016–1.287, *p* = 0.026), and with a history of ETOH (OR = 2.109, 95% CI, 1.724–2.580, *p* < 0.001) were more likely to receive ChEIs, whereas older women (OR = 0.976, 95% CI, 0.968–0.983, *p* < 0.001) were less likely to receive ChEI therapy. As presented in Figure 6, the predictive power of the logistic regression was moderately strong. The area under the curve (AUROC) is 0.688 (95% CI, 0.674–0.702, *p* < 0.001).

## 4. Discussion

In this study, LOAD is defined as the onset of AD after 65 years of age, and this description is used in other studies [26,45]. In general, AD has a deceiving and gradual onset making it occasionally difficult to distinguish from an age-related decline in the commencement of the disease [46]. The cut-off age of >65 years is subjective and may not associated any biological criteria; rather a social factor such as the traditional retirement age of 65 has been used as the cut-off age for LOAD [36,46]. The current study evaluated gender differences in LOAD patients treated with ChEI therapy. In the univariate analysis, our findings reveal that more women patients treated with ChEIs therapy received galantamine, while men with ChEI therapy were more likely to also receive SGAs including aripiprazole, and SSRIs such as citalopram, escitalopram, memantine, and buspirone. SSRIs are antidepressant agents that may delay the onset of AD [18]. As AD progresses, patients may become agitated, aggressive, or experience psychosis. SGA are used to treat these behavioral and psychological symptoms associated with this disease [47]. Similarly, buspirone is an anxiolytic medication commonly used to treat AD. In addition, memantine is an N-methyl-D-aspartate (NDMA) receptor regulates glutamate, which is the primary excitatory neurotransmitter in the brain [48,49].

Older and fewer men Caucasians with ChEI therapy presented with a history of ETOH. Few of the men patients who were treated with SGAs and SSRIs such as citalopram, escitalopram, memantine, and buspirone also received ChEI therapy. In addition, we found that fewer older Caucasians with a history of ETOH, were treated with SSRIs such as escitalopram and memantine also received ChEI therapy. In the adjusted analysis for the whole patients with LOAD, older patients were not likely to be treated with ChEI therapy, while those with a history of ETOH that received SSRIs including memantine were more likely to be treated with ChEI therapy. For the men patients, the effect of age which was associated with not being treated with ChEI therapy in the whole LOAD population were sustained in the men LOAD population, following an adjusted analysis. In addition, the effect of ETOH and SSRIs including memantine, citalopram, and buspirone, which were significant in the univariate analysis, were sustained in adjusted analysis and associated with ChEI therapy.

Older women LOAD patients were less likely to receive ChEI therapy. On the other hand, African American women with LOAD and a history of ETOH and taking SSRIs such as memantine were more likely to be treated with ChEI therapy. In general, while there were similarities in the effect of increasing age being less likely to be associated with treatment with ChEI therapy, and memantine being associated with ChEI therapy for both men and women patients, there were also specific differences. For example, while citalopram and buspirone were associated with ChEI therapy for the men patients, African American women with a history of ETOH and taking SSRIs were more likely to be treated with ChEI therapy. In general, the cholinergic hypothesis in AD is thought to be associated with a decrease in acetylcholine resulting in symptoms, and this represents the basis for the symptomatic treatment of AD [50]. Previous strategies, including the focus on agonists of the preserved postsynaptic muscarinic receptors such as choline, pilocarpine, and precursors of acetylcholine such as phosphatydylcholine have not been very successful due to severe side effects and discouraging trial results [51]. This provides a rationale for the new focus on the use of cholinomimetics which are known to imitate action of endogenously released acetylcholine [52]. Moreover, combining cholinomimetics, antipsychotics and memantine is known to have a modest effect on cognition [53]. For example, a combination of memantine and AChE inhibitors, in particular galantamine, show a significant effect in cognitive impairment because of their complementary pharmacological effects [54]. Our finding that a combined treatment using SSRIs with memantine or that SSRIs including memantine, citalopram, and buspirone were more likely to be treated with ChEI therapy supports the synergistic interaction between ChEI such as galantamine and memantine and SSRIs on cognition of AD patients [55].

In our finding, citalopram and buspirone were associated with ChEI therapy for the men patients, while African American women with a history of ETOH and taking SSRIs were more likely to be treated with ChEI therapy. A short-term positive cognitive response to ChEIs in men when compared with women was reported for tacrine and galantamine [37,38], and this was linked to the role of sex hormones in AD [39], and larger cerebral hemispheres were observed in men [40]. It is also possible that the existing explanation on the role of sex hormones in AD [39], and hemispheres differences reported in men and women may play a major role in a possible gender difference [40].

AD is known to reduce life expectancy in untreated conditions, especially in people affected at younger ages [56]. Whether ChEI treatment has a positive or negative effect on increasing age is not very clear, as the few AD studies that investigated this association reported inconsistent results [57,58]. For example, a mean survival age of 5.80 years following AD diagnosis and the commencement of ChEI therapy was comparable to that of patients without treatment [58,59,60]. However, the survival rate after 2 years was higher in the ChEI-treated group compared with the untreated group [61]. Another study that compared patients treated with untreated AD conditions found no difference between these groups in patients aged < 85 years; however, a longer lifespan was observed among the oldest ChEI-treated participants [41]. The aforementioned association between age and ChEI-treated patients was not observed in another study [56]. Taken together, these studies support our current findings that increasing age was not associated with ChEI therapy in our men and women LOAD AD population. It is possible that older people are receiving more aggressive pharmacological therapies rather than ChEI against other co-morbid disorders [61]. It is also possible that older individuals may present less aggressive forms of AD, and this could lead to the early detection of the AD, diagnosis and ChEI therapy at an earlier stage [36,62]. These factors might imply a lower mortality rate with increasing age in older AD patients in our LOAD population.

The neuropsychiatric symptoms of AD are associated with abnormalities in the serotonergic, noradrenergic, cholinergic, and dopaminergic neurotransmitter systems [63]. The hypothesis is that agitation in AD is caused by disruption in the afferent brain monoamine system resulting in the degeneration of the serotonergic pathways disrupting the homeostasis of the serotonergic–dopaminergic axis [64]. Studies on buspirone and citalopram found that both interventions reduced agitation and were better tolerated in AD patients [65,66]. Therefore, SSRIs such as citalopram and buspirone offer alternatives for the treatment of atypical antipsychotics where the side effect symptoms counterbalance the potential benefits [67]. Our finding that men AD dementia with LOAD were more likely to be treated with citalopram and buspirone is supported by existing studies that in AD patients with dementia, men with agitation are more likely to be treated with antipsychotics than are women with agitation [7,68]. Therefore, our current finding reveals the use of citalopram and buspirone for the treatment of men with AD dementia that co-occur with agitation in a population of LOAD patients.

The clinical manifestation of AD may differ for African Americans compared to non-Hispanic whites, in that the former often present with an earlier age of onset and exhibit greater severity of symptoms at the time of presentation [69]. A growing body of evidence suggests that the prevalence of AD may be three times higher in older African Americans than in older non-Hispanic whites [70,71]. While African Americans with AD present with a slower functional decline [72] and longer survival rates [73], our finding indicates that women African American AD patients are more likely to be treated with ChEI therapy. This finding may lay the foundation for understanding pharmacological factors to manage LOAD among African Americans and other minority groups. Our finding that men and women LOAD patients treated ChEI therapy also received memantine is supported by existing studies [74], indicating that memantine is beneficial for AD patients with moderate-to-severe AD. Since ChEIs may be used for mild, moderate, and severe disease [75], adding memantine for moderate to severe symptoms in LOAD may together work better than the ChEI drugs on their own.

## 5. Limitations

As with all retrospective studies, there are a number of potential limitations that must be kept in mind in interpreting the study results. The retrospective data were from a single institution. For this reason, the results cannot be extrapolated to other institutions. Since the study is a retrospective data collection of electronic medical records, there is tendency for human error as data from some study candidates may have been excluded which could have altered the results. In addition, the retrospective nature of this study did not include data on reasons why patients were presented for care. Moreover, data on the systematic evaluation of behavioral disorders were not available, since issues in behavioral disorders and the drugs used for treatment are usually considered for the prescription of ChEIs. In addition, data on MMSE and CDR were not available to determine disease progression and/or behavioral alterations, while information on the duration of ChEIs was not included the data base. The problem of distinguishing age-associated decline following the commencement of the disease made individual’s age at the onset of symptoms difficult to estimate accurately. Due to this fact, there is a possibility that some patients were misclassified as either EAOD or LOAD. In addition, data for a young group of populations suffering with AD and who received ChEI therapy were not included in our analysis. All of our subgroup analyses were predetermined, and our analyses were repeated several times to eliminate the possibility of type 1 statistical errors. While this is a single study, the demonstration of consistent gender disparities in the demographic and pharmacological characteristics increases the generalizability of our findings.

## 6. Conclusions

In the current study, we observed similarities in men and women LOAD patients in the effect of increasing age being associated with not being treated with ChEI therapy, and memantine was associated with ChEI therapy. We also observed differences in that citalopram and buspirone were associated with ChEI therapy for the men patients, while women African American patients were more likely to be treated with ChEI therapy. This finding highlights the importance for clinicians to optimize the ChEI and other pharmacological agents in AD, regardless of gender, to improve treatment effectiveness.

## Figures and Tables

**Table 1:** Comparison of Demographic and Clinical Characteristics of Late-Onset Alzheimer’s Disease Patients.

Characteristic	Men	Women	
Number of Patients	2949	6341	*p*-Value
Age Group: No. (%)			
60–69	104 (1.6)	32 (1.1)	0.007 *^a^
70–79	1076 (17.0)	562 (19.1)	
≥80	5161 (81.4)	2355 (79.9)	
Mean ± SD	85.59 ± 7.15	86.60 ± 7.51	<0.001 *^b^
Race: No (%)			
White	5218 (82.3)	2590 (87.8)	<0.001 *^a^
Black	790 (12.5)	246 (8.3)	
Other	333 (5.3)	113 (3.8)	
Hispanic Ethnicity: No. (%)	148 (2.3)	31 (1.1)	<0.001 *^a^
ETOH	680 (10.9)	526 (18.2)	<0.001 *^a^
Tobacco	2019 (32.5)	1879 (65.5)	<0.001 *^a^
Medications			
Central acetylcholinesterase inhibitor	3983 (62.8)	1902 (64.5)	0.117
Donepezil	3456 (54.5)	1657 (56.2)	0.128
Galantamine	77 (1.2)	62 (2.1)	0.001 *^a^
Rivastigmine	782 (12.3)	342 (11.6)	0.312
Second Generation Antipsychotic	1017 (16.0)	470 (15.9)	0.902
Aripiprazole	175 (2.8)	34 (1.2)	<0.001 *
Olanzapine	267 (4.2)	131 (4.4)	0.608
Risperidone	681 (10.7)	318 (10.8)	0.950
Selective Serotonin Receptor Inhibitor	2260 (35.6)	806 (27.3)	<0.001 *^a^
Citalopram	819 (12.9)	286 (9.7)	<0.001 *^a^
Escitalopram	1489 (23.5)	529 (17.9)	<0.001 *^a^
Paroxetine	0 (0.0)	0 (0.0)	
Memantine	2783 (43.9)	1385 (47.0)	0.006 *^a^
Trazadone	0 (0.0)	0 (0.0)	
Trazadone	501 (7.9)	170 (5.8)	<0.001 *^a^
Valproate	0 (0.0)	0 (0.0)	

Notes: ^a^ Pearson’s chi-squared test; ^b^ Student’s T test; * *p*-value < 0.05.

**Table 2:** Comparison of Demographics and Clinical Characteristics of Late-Onset Alzheimer’s Disease Patients who were taking a Central Acetylcholinesterase Inhibitor dependent on Gender.

	Men			Women		
Characteristic	No CAI	CAI		No CAI	CAI	
Number of Patients	1047	1902	*p*-Value	2358	3982	*p*-Value
Age Group: No. (%)						
60–69	2 (0.2)	30 (1.6)	0.002 *^a^	25 (1.1)	79 (2.0)	<0.001 *^a^
70–79	198 (18.9)	364 (19.1)		359 (15.2)	717 (18.0)	
≥ 80	847 (80.9)	1508 (79.3)		1974 (83.7)	3187 (80.0)	
Mean ± SD	86.21 ± 7.24	85.26 ± 7.07	0.001 *^b^	87.85 ± 7.88	85.86 ± 7.18	<0.001 *^b^
Race: No (%)						
White	933 (89.1)	1657 (87.1)	0.136	1979 (83.9)	3239 (81.3)	0.001 *^a^
Black	73 (7.0)	173 (9.1)		247 (10.5)	543 (13.6)	
Other	41 (3.9)	72 (3.8)		132 (5.6)	201 (5.0)	
Hispanic Ethnicity: No. (%)	12 (1.2)	19 (1.0)	0.716	48 (2.1)	100 (2.5)	0.237
ETOH	154 (15.1)	372 (19.9)	0.002 *^a^	154 (6.7)	526 (13.3)	<0.001 *^a^
Tobacco	674 (67.0)	1205 (64.7)	0.213	744 (32.5)	1275 (32.5)	0.965
Medications						
Second Generation Antipsychotic	140 (13.4)	330 (17.4)	0.005 *^a^	386 (16.4)	631 (15.8)	0.580
Aripiprazole	8 (0.8)	26 (1.4)	0.142	70 (3.0)	105 (2.6)	0.435
Olanzapine	39 (3.7)	92 (4.8)	0.161	100 (4.2)	167 (4.2)	0.927
Risperidone	100 (9.6)	218 (11.5)	0.109	251 (10.6)	430 (10.8)	0.851
Selective Serotonin Receptor Inhibitor	193 (18.4)	613 (32.3)	<0.001 *^a^	753 (31.9)	1507 (37.8)	<0.001 *^a^
Citalopram	32 (3.1)	254 (13.4)	<0.001 *^a^	288 (12.2)	531 (13.3)	0.200
Escitalopram	161 (15.4)	368 (19.3)	0.007 *^a^	487 (20.7)	1002 (25.2)	<0.001 *^a^
Paroxetine	0 (0.0)	0 (0.0)		0 (0.0)	0 (0.0)	
Memantine	254 (24.3)	1131 (59.5)	<0.001 *^a^	590 (25.0)	2193 (55.1)	<0.001 *^a^
Trazadone	0 (0.0)	0 (0.0)		0 (0.0)	0 (0.0)	
Buspirone	33 (3.2)	137 (7.2)	<0.001 *^a^	192 (8.1)	309 (7.8)	0.583
Valproate	0 (0.0)	0 (0.0)		0 (0.0)	0 (0.0)	

Notes: ^a^ Pearson’s chi-squared test; ^b^ Student’s T test; * *p*-value < 0.05.

## Data Availability

The retrospective datasets are available by request from the corresponding author of this manuscript.

## Abbreviations

AD: Alzheimer’s disease; AChE: Acetylcholinesterase inhibitors; IRB: Institutional Review Board; APOE: Apolipoprotein E; CDR; clinical dementia rating; ChEI: Cholinesterase inhibitor; EOAD: Early-onset Alzheimer’s disease; LOAD: Late-onset Alzheimer’s disease; NSAIDs; non-steroidal anti-inflammatory drugs; NMDA: N-methyl-D-aspartate; ETOH: Ethanol; MMSE: mini mental exam; SSRIs; Selective Serotonin Reuptake Inhibitor; *AUROC:* Area Under the Receiver Operating Characteristics: ROC; receiver operating characteristic curve; OR: Odd ratio; SGA: second generation antipsychotics; VIF: Variance Inflation factor.
